# Supplementary material for: Downregulation of BUD31 Promotes Prostate Cancer Cell Proliferation and Migration via Activation of p-AKT and Vimentin In Vitro
Source: Int J Mol Sci. 2023 Mar 23;24(7):6055. doi: 10.3390/ijms24076055 (PMC10094631; doi:10.3390/ijms24076055)
Supplement: Supplementary file 1 [file ijms-24-06055-s001.zip › ijms-2262371-supplementary.pdf]

Supplementary Materials

Supplementary Table S1. List of Antibodies.

No	Antibody	Catalog number	Supplier	Western Blot Dilution
1	Anti-BUD31 antibody (WB)	11798-1-AP	Protein tech	1:1000
2	Mouse anti-GAPDH antibody	60004-1-Ig	Protein tech	1:1000
3	Anti-Cyclin B1 antibody	Y106	Abcam	1:1000
4	Anti-P-PDK antibody	S241	Cell Signaling	1:1000
5	Anti-Vimentin antibody	Sc-6260	Santa Cruz	1:1000
6	Anti-N-Cadherin antibody	Sc-393933	Santa Cruz	1:1000
7	Anti-P-Akt antibody	4060P	Cell Signaling	1:1000
8	Anti-Akt antibody	4691P	Cell Signaling	1:1000
9	Mouse anti-Rabbit IgG antibody	7076S	Cell Signaling	1:1000
10	Rabbit anti-Mouse IgG antibody	7074S	Cell Signaling	1:1000
11	Anti-GARS antibody (IHC)	HPA019097	Sigma-Aldrich	1:500
